# Tempering implementation optimism: distinguishing between efficacy and effectiveness in implementation research

**DOI:** 10.1186/s43058-025-00781-2

**Published:** 2025-08-23

**Authors:** Per Nilsen, Jeanette Wassar Kirk, Katarina Ulfsdotter Gunnarsson, Kristin Thomas

**Affiliations:** 1 Department of Health, Medicine and Caring Sciences, Linköping University, Linköping, Sweden; 2 Faculty of Medicine and Health Sciences, Halmstad University, Halmstad, Sweden; 3 Department of Clinical Research, Hvidovre University Hospital, Copenhagen, Denmark; 4 Department of Health and Social Context, National Institute of Public Health, University of Southern Denmark, Odense, Denmark

**Keywords:** Efficacy, Effectiveness, Strategies

## Abstract

**Background:**

The distinction between efficacy (performance under ideal conditions) and effectiveness (performance in real-world settings) is well established in intervention research. Intervention effectiveness is often used as a proxy for implementation readiness. However, relying on this assumption can lead to overly optimistic expectations about real-world outcomes if the complexities of routine practice settings are not adequately considered.

**Main body:**

This paper introduces the distinction between implementation efficacy (implementation strategy performance under controlled or highly supported conditions) and implementation effectiveness (performance under typical, resource-constrained settings). We argue that the efficacy–effectiveness distinction is as critical for implementation research as it is for intervention research. Recognizing and systematically operationalizing this distinction can sharpen conceptual clarity, strengthen research design and enhance the relevance and generalizability of findings for real-world application.

Yet despite its importance, this distinction is rarely made explicit in implementation studies. Research often fails to specify the conditions under which implementation strategies are investigated; studies can vary widely in how closely they reflect routine practice. Compounding this issue, economic evaluations remain uncommon in implementation research. However, without systematic assessment of resource use, it is difficult to determine whether reported implementation outcomes have been achieved through contextually feasible strategies or through intensive supports, such as dedicated staffing, external facilitation, or financial incentives, which are rarely available in everyday practice.

To address this gap, we propose adapting the PRECIS-2 (Pragmatic Explanatory Continuum Indicator Summary 2) framework from clinical trials into an “Implementation PRECIS” tool. An adapted version of PRECIS-2 for implementation research could offer a systematic way to describe the extent to which a study reflects idealized conditions versus real-world practice.

**Conclusion:**

Clarifying whether implementation strategies are studied under efficacy-like or effectiveness-like conditions enhances research design, interpretation, and communication with stakeholders. It also supports informed decisions about replication and scale-up. By embracing this distinction, implementation research can temper overly optimistic assumptions, better reflect real-world constraints, and contribute more meaningfully to evidence-based practice. We argue that making this distinction explicit is a necessary step toward a more pragmatic and transparent science of implementation.

Contributions to the literature

- This debate paper introduces a clear distinction between implementation efficacy (ideal conditions) and implementation effectiveness (real-world conditions), bringing conceptual clarity to a commonly conflated area in the literature.
- The paper cautions against assuming that strategies proven under supportive conditions will scale successfully, calling for more detailed reporting and critical appraisal of study contexts.
- The importance of economic evaluation in implementation studies is emphasized, addressing a known gap in implementation research.
- An "Implementation PRECIS" tool is proposed by adapting the PRECIS-2 framework to implementation research, helping to assess how closely studies reflect real-world practice and improving design transparency and comparability.

## Introduction

Distinguishing between efficacy and effectiveness is essential for understanding how clinical interventions are developed, tested, and translated into practice. Efficacy refers to whether an intervention works under ideal, highly controlled conditions, whereas effectiveness concerns the intervention’s ability to achieve intended outcomes in real-world, everyday settings [1, 2]. This distinction has important implications for how research findings are interpreted, how policies are formed, and how interventions are scaled for broader implementation.

In implementation research, intervention effectiveness is usually used as a proxy for implementation readiness [3]. For example, Curran’s influential Hybrid Design taxonomy [4] implicitly assumes that interventions demonstrating both efficacy and effectiveness are ready for implementation. However, such assumptions may lead to overly optimistic expectations about real-world performance if they fail to account for the complexities of routine practice settings. This concern is underscored by the “efficacy–effectiveness gap”, which highlights the often substantial discrepancy between an intervention’s theoretical potential under ideal conditions and its real-world impact [5].

Implementation strategies are a form of intervention, but unlike clinical interventions, where the unit of analysis is typically patients or populations, these interventions focus on implementers such as providers, teams, units, or organizations [6]. An intervention is a deliberate action, strategy, or programme intended to bring about a desirable change [7]. However, implementation research often fails to specify the conditions under which implementation strategies are investigated; studies can vary widely in how closely they reflect routine practice [8–10]. Thus, the efficacy–effectiveness distinction is rarely made explicit in implementation studies. Compounding this issue, economic evaluations are uncommon in implementation research [11–13].

However, without systematic assessment of the conditions under which implementation occurs, including resource use, staffing levels, external facilitation and financial incentives, it is difficult to determine whether reported implementation outcomes were achieved through feasible strategies or relied on intensive supports that are rarely available in everyday practice. Although many implementation frameworks and models outline stages of implementation [14, 15], they typically focus on temporal progression rather than on the spectrum of conditions ranging from tightly controlled, efficacy-like conditions to the variability of real-world, effectiveness-like conditions.

This paper argues that the efficacy–effectiveness distinction is as critical for implementation research as it is for intervention research. Recognizing and systematically operationalizing this distinction can sharpen conceptual clarity, strengthen research design and enhance the relevance and generalizability of findings for real-world application.

## Understanding the efficacy–effectiveness distinction

### Efficacy vs. effectiveness and similar concepts

It is crucial to distinguish between efficacy and effectiveness to understand how interventions move from controlled trials to real-world applications [16]. Efficacy studies, often randomized controlled trials, assess whether an intervention can produce desired outcomes under ideal conditions, with tightly controlled environments and carefully selected participants [17]. For example, a drug might be tested in a hospital with precise dosing and professional oversight, aiming to answer the question “can it work?” [18].

In contrast, effectiveness studies evaluate how interventions perform in everyday clinical settings with more diverse populations, varying fidelity, and complex contextual factors [18]. Using the same drug example, an effectiveness trial would assess outcomes among patients with comorbidities, differing lifestyles, and typical medical supervision, addressing the question “does it work in practice?” [18].

Flay [19] formalized this distinction: efficacy trials test interventions under optimal conditions, whereas effectiveness trials examine outcomes in real-world contexts. The former ensures internal validity through standardization and homogeneous populations; the latter prioritizes external validity by embracing real-world variability. According to Flay [19], demonstrating efficacy is a prerequisite for meaningful effectiveness testing and, ultimately, implementation.

This distinction aligns with explanatory versus pragmatic trials [20]. Whereas explanatory trials maximize internal validity, pragmatic trials are designed to inform real-world decision-making. Similarly, Weisz et al. [21] and Shadish et al. [22] contrast research therapies, which may not reflect clinical realities, with clinic therapies, which mirror routine practice and are more readily implementable.

Table 1 summarizes five key aspects of intervention studies: setting, goal, population, validity focus, and core question. For each aspect, the table outlines the distinguishing characteristics of efficacy and effectiveness studies, highlighting the differences between these two concepts.

**Table 1:** Key characteristics of intervention efficacy and effectiveness studies

Aspect	Efficacy	Effectiveness
Setting	Controlled, usually a research environment	Real-world setting
Goal	Ideal performance	Practical performance
Population	Homogeneous, carefully selected	Diverse, general population
Validity focus	High internal, low external validity	Lower internal, higher external validity
Core question	Can it work?	Does it work in practice?

### Reasons for the efficacy–effectiveness divergence

The efficacy and effectiveness of an intervention depend not only on its core features but also on the context in which it is implemented, the providers delivering it and the patients or population receiving it. Efficacy studies are typically conducted in controlled settings with intensive oversight to ensure the intervention is delivered as intended, with fidelity. In contrast, effectiveness studies take place in real-world environments where providers may face time constraints, varying levels of training, and competing priorities. These conditions can reduce fidelity, potentially weakening the intervention’s impact [19].

Differences in target populations also contribute to the efficacy-effectiveness gap. Efficacy trials often involve highly selected, motivated participants with few complicating factors, whereas effectiveness studies aim to reflect the broader, more complex realities of clinical practice. Contextual variation further influences outcomes; interventions tested in resource-rich academic centers may perform differently when applied in under-resourced or high-pressure settings, where delivery and uptake are shaped by the surrounding environment [16].

As with clinical interventions, the efficacy and effectiveness of an implementation strategy depend not only on the strategy itself but also on the context in which it is deployed, the implementers delivering it (e.g. providers or organizations), and the setting’s operational capacity. Studies of implementation efficacy are typically conducted under ideal or highly supported conditions, with dedicated facilitation, training, funding, or researcher involvement, to ensure the strategy is delivered as intended. In contrast, implementation effectiveness is evaluated in routine practice settings, where implementers often face limited time, minimal support, and competing demands. These constraints can reduce fidelity and diminish impact [8].

The characteristics of the implementers, e.g. the organization where the implementation occurs, also influence outcomes. Efficacy studies may involve motivated, well-resourced organizations predisposed to adopt change, whereas effectiveness studies reflect more typical settings with varying levels of readiness, leadership, and capacity. Contextual factors, such as professional cultures, work climate and infrastructure, further shape outcomes. Strategies that succeed in high-capacity environments may underperform in overstretched systems, underscoring the need to distinguish what works under optimal versus routine conditions [15].

### Applying efficacy–effectiveness in implementation research

Intervention research often assumes that an intervention is ready for implementation and large-scale dissemination once effectiveness is demonstrated [5]. From this perspective, effectiveness is treated as a green light to scale interventions across diverse settings, fueling much of the momentum behind evidence-based medicine and practice [23]. In reality, however, demonstrated effectiveness does not guarantee success in routine practice. Even effectiveness studies often involve additional resources, specialized training, enhanced monitoring, or participant incentives, conditions rarely replicated in everyday practice [24]. Furthermore, individuals’ behavior can change simply because they know they are being studied; these research participation effects can limit generalizability [25].

Implementation research often encompasses studies conducted under both idealized and routine practice conditions, without clearly distinguishing between the two [4]. Many studies use multiple strategies to address barriers to implementation [6, 26]. These strategies may create highly supportive environments, in many ways resembling intervention efficacy studies. Although such support may improve short-term implementation outcomes, it often fails to reflect the constraints of resource-limited or high-pressure settings.

In contrast, some implementation studies intentionally restrict external support, studying “low intensity” strategies that rely on existing infrastructure and staff to reflect typical practice conditions [27]. These designs better approximate real-world conditions. Both types of studies are grouped under the broad label of “implementation research,” often without specifying the level of support or study conditions involved.

This variation suggests a useful distinction: implementation efficacy refers to whether an implementation strategy works under idealized conditions, e.g., with external facilitation, dedicated funding, or researcher oversight, whereas implementation effectiveness examines how a strategy performs under routine conditions, with standard staffing and minimal added support. This mirrors the conceptual divide between intervention efficacy and effectiveness.

Table 2 applies the five aspects outlined in Table 1 (setting, goal, population, validity focus, and core question) to implementation studies, illustrating characteristics of implementation efficacy and implementation effectiveness studies.

**Table 2:** Examples of characteristics of implementation efficacy and effectiveness studies

Aspect	Implementation efficacy	Implementation effectiveness
Setting	Controlled or supportive setting, e.g., external facilitation, staff training and additional funding	Routine setting, with existing staff, infrastructure, and minimal extra support
Goal	Test if an implementation strategy can succeed under ideal conditions	Test if an implementation strategy does succeed under real-world conditions
Population	Select implementers (organizations, units, teams, providers, etc.), often with greater readiness	Diverse implementers with variable readiness and typical operational pressures
Validity focus	High internal validity, focused on isolating strategy effects	Higher external validity focused on generalizability across real-world contexts
Core question	Can this implementation strategy work under optimal conditions?	Does this implementation strategy work under routine conditions?

### Positioning implementation studies along an efficacy–effectiveness continuum

To operationalize the distinction between implementation efficacy and implementation effectiveness, we propose that implementation researchers should develop an "Implementation PRECIS" tool, analogous to the PRECIS-2 (Pragmatic Explanatory Continuum Indicator Summary 2) framework used in clinical trials [28]. By enhancing the transparency of study design choices, this tool would support researchers in clearly situating their implementation study along the efficacy–effectiveness continuum.

PRECIS-2 assesses studies across nine domains to determine how tightly controlled (explanatory) or real-world (pragmatic) a study is. Table 3 presents the domains of PRECIS-2. Each domain is rated on a continuum, providing a structured way to visualize the degree to which a study mirrors routine practice versus idealized research conditions. PRECIS-2 thus helps clarify the intended purpose of a study: whether it seeks to establish causal efficacy under optimal circumstances or to test effectiveness in the messy reality of everyday care [28].

**Table 3:** The nine domains of PRECIS-2 [27]

PRECIS-2 domain	Brief description
Eligibility	How similar are the trial participants to typical patients?
Recruitment	How are participants approached: routine care versus special efforts?
Setting	Does the setting reflect everyday clinical environments?
Organization	Does it use typical resources and infrastructure?
Flexibility (delivery)	How strictly is the intervention delivered?
Flexibility (fidelity)	How closely must participants follow the intervention?
Follow-up	Is monitoring the same as in usual care?
Primary outcome	Is the outcome meaningful to patients and decision-makers?
Primary analysis	Does the analysis reflect real-world use (e.g., intention-to-treat)?

Although PRECIS-2 was developed for clinical trials, its core logic could also be adapted to categorize implementation studies. A similar continuum exists in implementation research. Some studies test implementation strategies under highly supportive, efficacy-like conditions (e.g., using external facilitation, training, and dedicated funding); others study implementation under routine, effectiveness-like conditions using standard resources and typical workflows.

PRECIS-2 can be adapted for implementation research to offer a systematic way to describe the extent to which a study reflects idealized conditions versus real-world practice. Table 4 illustrates how the nine domains of PRECIS-2 could be adapted for implementation research, showing how each domain might be translated to fit implementation contexts instead of clinical interventions. This adaptation could serve as the foundation for an “Implementation PRECIS” tool, supporting researchers in designing, assessing, and reporting implementation studies with greater transparency, contextual nuance, and practical relevance.

**Table 4:** Exemplifying how PRECIS-2 could be applied in implementation research to assess and describe the degree to which an implementation study aligns with idealized versus real-world conditions

Original domain	Implementation translation
Eligibility	Which implementers (e.g., organizations, units, teams, providers) are included in the study? Are they representative of typical real-world settings, or are they purposively selected (e.g., only including highly motivated or well-resourced hospital clinics)?
Recruitment	How are implementation sites or providers recruited? Through routine channels or intensive outreach? Is participation entirely voluntary or are there financial or other incentives involved?
Setting	What kind of settings are included? What are the internal and external conditions? Do they reflect the diversity and complexity of real-world practice (e.g., urban versus rural primary care centers, small versus large hospital clinics)?
Organization	What supportive strategy (e.g., facilitation or training) is used for the implementation? Is additional support such as resources, staffing, or infrastructure required, or is implementation executed within existing routines and organizational capacity?
Flexibility (delivery)	How strictly must the supportive implementation strategies be executed or delivered? Are sites and/or providers allowed to adapt to their local context, or is fidelity enforced?
Flexibility (fidelity)	How closely are implementers expected to follow the implementation strategy protocols? Is variation tolerated, or is fidelity monitored and enforced?
Follow-up	How is implementation progress monitored over time? Is there intensive data collection or oversight, or light-touch tracking typical of routine practice?
Primary outcome	Are the outcomes meaningful to the implementers? Do outcomes reflect what matters to the implementers (e.g., workflow integration, sustainability), or are they researcher-driven?
Primary analysis	Is the analysis reflective of real-world implementation? Does it include all sites or providers as they implemented or only those with high fidelity?

### Benefits of the distinction between implementation efficacy and effectiveness

Distinguishing between implementation efficacy and implementation effectiveness offers several benefits for advancing the field of implementation research. First, it enhances conceptual clarity by providing precise terminology that helps researchers articulate and define the nature of their studies and the conditions under which they are conducted. This precision improves academic discourse and facilitates more transparent communication with stakeholders by linking terms to specific levels of support, structure, and control.

Second, embracing this distinction supports scalability and real-world translation by giving policymakers and practitioners a clearer picture of what successful implementation requires beyond controlled studies. Without explicit acknowledgment of support levels, there is a risk of overestimating feasibility, leading to unrealistic expectations and inefficient resource use.

Third, the distinction allows for a more accurate assessment of implementation strategy performance. Some strategies may appear highly effective primarily due to intensive, possibly unsustainable, support. Distinguishing implementation efficacy from effectiveness helps identify which strategies are robust and which are context-dependent, enabling more meaningful comparisons and informed decisions about replication and scale-up.

Fourth, it strengthens economic evaluations by clarifying the resource and infrastructure requirements for successful implementation. This enables more realistic assessments of cost-effectiveness and supports the prioritization of strategies that are both impactful and economically sustainable in real-world settings.

Fifth, applying a PRECIS-like framework to implementation studies offers a structured approach for addressing these challenges. It improves transparency by explicitly stating the level of support required, sharpens interpretation of findings, and fosters more meaningful evidence synthesis. Most importantly, it distinguishes between success under optimal conditions (implementation efficacy) and routine practice (implementation effectiveness), providing a stronger basis for evaluating scalability. As the field matures, tools such as PRECIS-2 offer valuable guidance for improving research design and reporting.

## Discussion

In this paper, we have argued that the well-established efficacy–effectiveness distinction in intervention research should be systematically extended to implementation research, given that implementation strategies are a form of intervention, albeit with a different unit of analysis. Some may object that implementation is too context-dependent for such classifications. Although implementation is indeed shaped by factors such as culture, leadership, and organizational readiness, recognizing a continuum, from high- to low-support conditions, adds clarity without oversimplifying complexity.

Clarifying the distinction between implementation efficacy and effectiveness will require intentional effort. A critical step could be the creation of an "Implementation PRECIS" tool, drawing on inspiration from the PRECIS-2 framework used in clinical trials [28]. The tool should be flexible enough to accommodate the distinct needs of various users, including implementation practitioners and healthcare leaders. However, developing such a tool is challenging because studies often combine elements of both efficacy-like and effectiveness-like conditions. Despite these challenges, such a tool could help position studies along a continuum from highly controlled, resource-intensive conditions to routine practice.

Capturing the distinction between efficacy and effectiveness studies improves transparency, comparability, and interpretability of findings. This is crucial not only for academia, but also for stakeholders such as implementation practitioners, policy-makers, funders and healthcare leaders to assess feasibility and scalability of implementation efforts.

In conclusion, although implementation research has made significant strides, it remains susceptible to overly optimistic assumptions about real-world applicability. Advancing a cumulative science in implementation research depends on distinguishing whether implementation strategies are tested under ideal or real-world conditions. By revisiting and emphasizing the efficacy–effectiveness distinction, the field can achieve greater conceptual clarity in reporting. This approach will help temper implementation optimism, improve research transparency, and support more informed and pragmatic decision-making across diverse settings, ultimately strengthening the foundation of evidence-based practice.

## Data Availability

Not applicable. No datasets were generated or analyzed during the current study.
